# HTLV Seroprevalence and Associated Factors Among Blood Donors in Shenzhen, China: A Cross-Sectional Study with Longitudinal Follow-Up, 2013–2022

**DOI:** 10.3390/v18101058

**Published:** 2026-09-24

**Authors:** Xiaoxuan Xu, Dian Wang, Guiwen Guan, Guochao Wei, Linfeng Wu, Heng Liu, Ran Li, Haobiao Wang, Tingting Chen, Fengmin Lu, Yuanye Xue, Xiangmei Chen, Jinfeng Zeng

**Affiliations:** 1 Department of Laboratory, Shenzhen Blood Center, Shenzhen 518035, China; 2 Department of Microbiology and Infectious Disease Research Center, School of Basic Medical Sciences, Peking University Health Science Center, Beijing 100191, China; 3 College of Veterinary Medicine, Northwest A&F University, Yangling, Xianyang 712199, China

**Keywords:** HTLV, blood donors, follow-up, seroprevalence, blood safety, epidemiological study

## Abstract

Background: Blood transfusion is a major route of Human T-lymphotropic virus (HTLV) transmission. In this study, we conducted a cross-sectional study of HTLV seroprevalence among blood donors in Shenzhen, with secondary investigations covering signal-to-cutoff (S/CO) predictive value, longitudinal follow-up, epidemiological characteristics, and possible familial clustering. Methods: Retrospective analysis was conducted on 512,200 Shenzhen Blood Center donors (2014–2022, with partial 2013 data). ELISA was utilized for initial HTLV screening, with MP, INNO-LIA and qPCR for confirmation. Confirmed cases received questionnaire follow-up and retesting. Some relatives of confirmed cases also received HTLV-1/2 testing. Results: Among 512,200 donors, 203 were screening-reactive, with 18 confirmed-positive cases (3.51 per 100,000 donors; 95% CI: 2.08–5.55) and 10 indeterminate cases. The screening-reactive rate declined significantly over the decade (*p* < 0.001), whereas the confirmed-positive rate remained low and showed no significant temporal trend (*p* = 0.363). Among initially screening-reactive donors, high S/CO values strongly predicted confirmatory positivity, whereas low-level reactivity was mostly transient. Questionnaire data were available for 9 of the 18 confirmed-positive donors (50.0%) and 51 control donors. In this exploratory analysis, only invasive medical procedures occurring in Fujian remained significantly associated with infection (adjusted *p* = 0.010). Two of the 10 tested family members were confirmed-positive, suggesting possible familial clustering. Conclusions: The confirmed prevalence of HTLV among Shenzhen blood donors was extremely low, and screening reactivity declined significantly over the ten-year period. Screening S/CO values strongly predicted confirmatory results. Fujian-related invasive medical procedures and possible familial clustering are exploratory findings that require confirmation in larger studies. Continued donor screening and blood safety surveillance remain warranted.

## 1. Introduction

Human T-lymphotropic virus (HTLV) is a retrovirus that mainly infects T-cells and can cause serious diseases [1,2]. To date, four subtypes (HTLV-1 to 4) have been identified, with HTLV-1 and HTLV-2 being the most common [1,3]. While most HTLV-1 carriers are asymptomatic, approximately 5–10% may develop adult T-cell leukemia or myelopathy. The pathogenicity of HTLV-2 remains unclear [4,5], and knowledge about HTLV-3 and HTLV-4 is limited [6,7]. Globally, an estimated 5–10 million people are infected with HTLV-1 [8], with high-prevalence regions including Japan, Africa, and parts of the Americas [2,9], though the actual number is likely an underestimate. There is currently no curative antiviral therapy for HTLV infection, although HTLV-associated diseases can be managed clinically.

HTLV is transmitted through direct contact with infected lymphocytes, primarily via breastfeeding, sexual contact (especially male-to-female transmission), or bloodborne routes such as transfusions, needle sharing, or contaminated injuries [10]. Blood transfusion represents a major transmission route for HTLV-1, whereas HTLV-2 is most frequently spread among people who inject drugs [10]. Given the absence of effective treatment, prevention remains essential for controlling HTLV spread.

Following Japan’s introduction of blood donor screening for HTLV in 1986 [11], many countries and regions—including North America, several European nations, Saudi Arabia, and Brazil—have established routine donor screening [9,12,13,14]. Screening strategies vary according to the local prevalence: in low-prevalence areas, donor questionnaires and targeted testing of individuals from high-risk areas are common; in high-prevalence regions, mandatory universal screening is typically implemented [15,16]. Notably, in sub-Saharan Africa, one of the major HTLV-1-endemic regions worldwide, routine donor screening remains largely absent, leaving the risk of transfusion-transmitted HTLV-1 insufficiently controlled [17,18]. Additionally, leukocyte filtration of donated blood has proven effective in reducing the risk of transfusion-transmitted HTLV [16].

In China, Fujian Province reports the highest prevalence of HTLV, at 36.24 per 100,000 population. Other provinces, such as Hunan, Zhejiang, and Beijing, also show rates exceeding 3 per 100,000, which are slightly elevated compared to most other regions [19]. Shenzhen, a major coastal city with a population of 17 million, faces potential HTLV transmission risks arising from population mobility and labor migration, which create epidemiological connectivity with areas of relatively high prevalence, including Fujian and Hunan. Despite these concerns, no systematic study has yet evaluated the actual risk or prevalence of HTLV in Shenzhen.

In this study, we analyzed HTLV-related data from the Shenzhen Blood Center (SZBC) between 2013 and 2022 and conducted follow-up surveys among HTLV-positive blood donors to explore potential transmission patterns. The primary objective of this study was to estimate HTLV seroprevalence among blood donors in Shenzhen; secondary objectives included evaluating the relationship between initial screening signal-to-cutoff (S/CO) values and confirmatory outcomes, exploring epidemiological factors associated with HTLV positivity, and investigating possible familial clustering. Our findings are expected to help refine blood donor screening protocols and recruitment strategies, thereby providing guidance for ensuring blood safety in megacities.

## 2. Materials and Methods

### 2.1. Study Population

This study included blood donors from Shenzhen Blood Center between 2014 and 2022, along with a subset of donors from 2013. As 2013 was the pilot year of HTLV screening in Shenzhen, all donors between 26 February and 22 April 2013 were tested, and all 2013 data in this study were derived from this period. All participants met the basic eligibility criteria of the Blood Donation Law of China, which require donors to be in good health, free from high-risk factors for blood-borne diseases, and aged between 18 and 65 years of age. Among the 512,200 donors, 315,511 were male and 196,689 were female; by age, 68,440 were ≤20 years, 219,778 were 21–30 years, 138,283 were 31–40 years, 69,915 were 41–50 years, and 15,784 were 51–65 years. For donors with multiple blood donations, only one record per individual was counted in the prevalence analysis. Donors with confirmed-positive or indeterminate HTLV-1/2 results before or during the study period were deferred from blood donation. Those with initial indeterminate results were required to undergo three follow-up tests within 3 to 12 months. Individuals with persistently indeterminate results were permanently deferred, while those testing negative were allowed to resume donation. This study was approved by the Ethics Committee of the Shenzhen Blood Center (SZBC-2020-R010). Written informed consent was obtained from all participating donors.

### 2.2. Initial Screening for HTLV-1/2 Infection

Initial screening for HTLV-1/2 antibodies was performed for all enrolled blood donors using either the Wantai HTLV-1/2 Antibody Detection Kit (Wantai BioPharm, Beijing, China) or the Sorin HTLV-1/2 Antibody Detection Kit (Diasorin S.p.A., Saluggia, Italy). Both kits utilize a double-antigen sandwich enzyme-linked immunosorbent assay (ELISA) for the simultaneous detection of antibodies against HTLV-1 and HTLV-2 in donor plasma samples. Samples with reactive results in the initial test were tested in duplicate using the same assay. Samples that were reactive in at least one of the duplicate tests were defined as initial screening-reactive samples.

### 2.3. Confirmatory Testing and Viral Typing for HTLV-1/2 Infection

Initial screening-reactive samples were sent to the National Center for Clinical Laboratories (NCCL) for confirmatory testing. Confirmatory testing was performed using the MP HTLV Blot 2.4 (MP Biomedicals, Singapore), Innogenetics line immunoassay (INNO-LIA HTLV I/II Score, Fujirebio, Tokyo, Japan), or quantitative PCR (qPCR) methods. During the ten-year study period, the MP assay was used for confirmation in the early years, and qPCR and INNO-LIA were subsequently introduced. The specific confirmatory assays used for each confirmed-positive or indeterminate donor are shown in Tables S1 and S2.

The INNO-LIA strips contain four non-type-specific antigens (gag p19 I/II and p24 I/II, env gp46 I/II and gp21 I/II) for confirming the presence of HTLV-1/2 antibodies, together with type-specific antigens for HTLV-1 (gag p19-I and env gp46-I) and for HTLV-2 (env gp46-II), enabling the differentiation between HTLV-1 and HTLV-2 infections. The MP HTLV Blot 2.4 utilizes nitrocellulose strips incorporated with HTLV-1 viral lysate and recombinant envelope antigens. Of the applied proteins, five antigens (rgp46-I, rgp46-II, GD21, p19, and p24) are used to confirm the presence of antibodies against HTLV-1/2; among these, the type-specific recombinant envelope proteins rgp46-I and rgp46-II are specific for HTLV-1 and HTLV-2, respectively, enabling the differentiation between HTLV-1 and HTLV-2 infections. However, because laboratory records were not standardized during the period when the MP assay was used, typing results were not documented; samples confirmed with this assay could therefore only be recorded as HTLV-1/2 positive (untyped). The results of INNO-LIA and MP assays were interpreted strictly according to the manufacturers’ package inserts.

The qPCR confirmatory testing was performed using a duplex TaqMan real-time PCR assay developed by Ji et al. [20]. Briefly, DNA was extracted from the whole blood of each blood sample, and the HTLV-1 pol region and the cellular gene RNase P (RPPH1) were co-amplified in a single tube using the primers and probes: (HTLV-1 pol F: 5′-CTAGCCCACTTGCAAACTATAGACCT-3′, HTLV-1 pol R: 5′-CGTAGTTACACTGCTGTGGGACAG-3′, HTLV-1 pol P 5′-AGACGCCTTTTTCCAAATCCCCTTACC-3′; RPPH1 F: 5′-CATGCCGTCCCGCGATATTGA-3′, RPPH1 R: 5′-GTTGATGACGTCAGCGTTCGAATTC-3′, RPPH1 P 5′-CTCCGAACCTCTCGCCCTGCC-3′). The probes were labeled by FAM (HTLV-1 pol) and VIC (RPPH1), respectively. A plasmid containing both the HTLV-1 of interest and a fragment of the RPPH1 gene [pcDNA3.1(-)-H1-pol-tax-RPPH1] was used to establish the standard curves for quantification. The normalized HTLV-1 proviral load (PVL) was calculated as the ratio of (HTLV-1 DNA average copy number/RPPH1 average copy number) × 2 × 100, expressed as the number of HTLV-1 copies per 100 cells. A sample was defined as qPCR-positive when a characteristic amplification curve of the HTLV-1 pol target was observed together with normal amplification of the RPPH1 internal control.

### 2.4. Follow-Up of Donors with Confirmed-Positive or Indeterminate HTLV-1/2 Results and Their Relatives

Following confirmatory testing, blood samples were collected for testing from donors with confirmed-positive or indeterminate results, as well as from their relatives. These follow-up samples were analyzed using the same serological screening and confirmatory workflows described previously (i.e., initial ELISA screening followed by MP, INNO-LIA, or qPCR confirmation). For the relatives of donors, follow-up was discontinued once a sample tested a confirmed-positive result; otherwise, a maximum of three follow-up visits were performed per individual.

### 2.5. Questionnaire Survey

A retrospective questionnaire survey was conducted among three groups: donors with confirmed HTLV-1/2 positive, donors with indeterminate results, and a randomly selected control group of confirmed-negative donors. The questionnaire was administered face-to-face by trained staff to ensure that participants understood each question. Nine of the 18 confirmed-positive donors completed the questionnaire. The 51 control questionnaires were voluntarily completed by blood donors randomly recruited within a one-month period, without matching or frequency matching on age, sex, year of donation, or other variables. For the sexual-behavior items related to multiple sexual partners, only 18 of the 51 controls provided responses. The questionnaire was obtained from the National Health Commission of the People’s Republic of China, and its full content is available in File S1. Based on the questionnaire responses, donors were classified into the following binary variables: Donors who reported any of the HTLV-endemic countries or regions listed in Question 16 were classified as “Travel to HTLV-endemic countries”. Donors who reported any invasive procedure in Question 17 (excluding blood donation) were classified as having a history of “Invasive medical procedures”. Donors were classified as having “Multiple sexual partners” if they reported two or more lifetime sexual partners in Question 20.3, or if the sum of marriages/cohabitations (Question 21.1) and commercial sexual partnerships (Question 22.1) was two or more. Donors who reported Fujian as their place of origin, birthplace, or place of upbringing in Question 3, who reported having visited Fujian in Question 15, or who reported having undergone relevant medical procedures in Fujian in Question 17 were classified as having a “Fujian-related background”. Within this composite variable, donors who reported Fujian as their place of origin or place of upbringing in Question 3 were classified as “Fujian origin/residence”, and donors who reported any invasive procedure in Question 17 (excluding blood donation) with the procedure place reported as Fujian were further classified as having “Invasive medical procedures” occurring in Fujian.

### 2.6. Statistical Analysis

Temporal trends were evaluated using Poisson regression with calendar year as the independent variable. For donors with repeated donations, S/CO measurements were averaged within each assay before analysis so that each donor contributed one observation per assay. As the S/CO values did not follow normal distributions, non-parametric tests were used. Differences among the three confirmatory groups were assessed using the Kruskal–Wallis test, followed by Dunn’s post hoc tests with Holm adjustment for multiple comparisons. The two groups were compared using a two-sided Mann–Whitney U test. For the questionnaire-based comparisons, associations between risk factors and confirmed HTLV infection were evaluated using chi-square tests with Yates’ continuity correction, and Fisher’s exact test was used for the Fujian-related comparisons; odds ratios (OR) with 95% confidence intervals (95% CI) were calculated using the Haldane–Anscombe correction. Adjusted *p* values were obtained using the Bonferroni correction, and post hoc power was calculated for each comparison at the observed effect size and group sizes using two-sided Fisher’s exact test (α = 0.05). Given the very low prevalence of confirmed HTLV infection in the source donor population, the OR provides a reasonable approximation of the relative association under the rare-disease assumption; ORs were therefore used as the measure of association in the exploratory questionnaire analysis. All statistical tests were two-sided, with a significance level of α = 0.05. All data management and statistical analyses were performed using R (version 4.4.2).

## 3. Results

### 3.1. Overall HTLV Screening and Confirmatory Outcomes Among Blood Donors in Shenzhen

Between 2013 and 2022, a total of 512,200 blood donors at the Shenzhen Blood Center were screened for antibodies against HTLV-1/2. The overall screening and confirmatory workflow is shown in Figure 1. Among all screened donors, 203 were repeatedly reactive in the initial ELISA screening, corresponding to an overall screening-reactive rate of 39.63 per 100,000 donors (95% CI: 34.37–45.48 per 100,000) (Table 1).

After confirmatory testing, 18 donors were confirmed as HTLV-1/2 positive, yielding an overall confirmed-positive rate of 3.51 per 100,000 donors (95% CI: 2.08–5.55 per 100,000). Among these confirmed-positive donors, 15 were identified as HTLV-1 infections, whereas the remaining 3 were untyped (HTLV-1/2). In addition, 10 initially reactive donors had indeterminate confirmatory results, corresponding to a rate of 1.95 per 100,000 donors (95% CI: 0.94–3.59 per 100,000). The remaining 175 initially reactive donors were confirmed-negative (Table 1).

Among the 203 initially reactive donors, only 18 were confirmed-positive, indicating that the positive predictive value of initial ELISA reactivity was 8.87% (95% CI: 5.68–13.58%) (Table 1). By contrast, 86.21% (175/203) of initially reactive donors were ultimately confirmed-negative and 4.93% (10/203) yielded indeterminate confirmatory results. These findings indicate that, in this low-prevalence blood donor population, initial ELISA reactivity alone had limited ability to identify true HTLV infection and therefore required confirmatory testing for accurate donor classification.

To assess temporal changes in HTLV infection among blood donors in Shenzhen, annual screening-reactive and confirmed-positive rates were calculated. Poisson regression analysis showed a significant negative association between calendar year and the screening-reactive rate (RR/year = 0.895, 95% CI: 0.848–0.944, *p* < 0.001) (Figure 2B), whereas the confirmed-positive rate also declined over time but did not reach statistical significance (RR/year = 0.921, 95% CI: 0.768–1.099, *p* = 0.363) (Figure 2C). These results indicate a significant decline in the screening-reactive rate among Shenzhen blood donors over the study period, whereas the confirmed-positive rate remained low and showed no significant temporal trend.

### 3.2. Relationship Between Initial Screening S/CO Values and Confirmatory Outcomes

To further evaluate whether initial screening signal-to-cutoff (S/CO) values were associated with confirmatory outcomes, S/CO values were analyzed for the 203 initially reactive donors. For donors who contributed more than one reactive specimen, the arithmetic mean of the S/CO values was used. Of these donors, 90 were screened with the Sorin assay and 116 with the Wantai assay; 3 donors contributed reactive specimens under both assays and are therefore counted in both assay groups.

Among the 90 donors screened with the Sorin assay, 11 were confirmed-positive (12.22%), 10 had indeterminate results (11.11%), and 69 were confirmed-negative (76.67%). The S/CO values were highest in the confirmed-positive group, with all confirmed-positive samples showing S/CO values greater than 10 (Table 2). For the Sorin assay, donor-level S/CO values differed significantly among the confirmed-positive, confirmed-negative, and indeterminate groups (Kruskal–Wallis H(2) = 36.20, *p* < 0.001). Dunn’s post hoc tests with Holm adjustment showed that S/CO values were significantly higher in the confirmed-positive group than in the confirmed-negative group (adjusted *p* < 0.001). The indeterminate group also had significantly higher S/CO values than the confirmed-negative group (adjusted *p* = 0.006). However, the difference between the confirmed-positive and indeterminate groups did not remain statistically significant after adjustment for multiple comparisons (adjusted *p* = 0.062) (Figure 3A).

Among the 116 donors screened with the Wantai assay, nine were confirmed-positive (7.76%) and 107 were confirmed-negative (92.24%). No indeterminate confirmatory results were observed among Wantai-reactive donors (Table 2). For the Wantai assay, confirmed-positive donors had significantly higher donor-level S/CO values than confirmed-negative donors (Mann–Whitney U = 5, *p* < 0.001, Figure 3B).

### 3.3. Longitudinal Follow-Up of Screening-Reactive Donors

Donors with confirmed-positive (*n* = 11), indeterminate (*n* = 10), and confirmed-negative (*n* = 41) results after initial reactivity were followed up through repeated sample collection and testing to determine their subsequent serological outcomes. Among 11 confirmed-positive donors who underwent follow-up testing, all remained reactive in subsequent screening and positive in confirmatory testing. Seven of these donors were initially screened using the Sorin assay and four using the Wantai assay. Across follow-up visits, their S/CO values remained consistently high and showed no obvious decline, supporting the stability of true HTLV seropositivity in confirmed-positive donors (Table 3, Figure 4A,B). Among the 10 donors with indeterminate confirmatory results, all were followed using the Sorin assay. Three donors became non-reactive during follow-up and were subsequently confirmed-negative, whereas seven donors remained screening-reactive and persistently indeterminate in confirmatory testing (Table 3, Figure 4E).

We also analyzed 41 donors who were initially screening-reactive but confirmed-negative and later returned for additional donation and HTLV retesting; of these, 26 became non-reactive during follow-up, whereas 15 remained screening-reactive but confirmed-negative. Among the 18 donors initially screened using the Sorin assay, 14 became non-reactive in subsequent screening, whereas 4 remained screening-reactive but confirmed-negative. Among the 23 donors initially screened using the Wantai assay, 12 became non-reactive, whereas 11 remained screening-reactive but confirmed-negative (Table 3, Figure 4C,D). Overall, these longitudinal patterns indicate that a substantial proportion of initially reactive but confirmed-negative donors subsequently became non-reactive.

Given that S/CO values near the cutoff were more frequently observed among confirmed-negative donors, we further examined whether donors with low-level reactivity were more likely to become non-reactive during follow-up. Among 62 donors who were followed up, 38 had initial low-level reactivity (S/CO values ≤ 2), including 5 with indeterminate confirmatory results and 33 with negative confirmatory results. During follow-up, 24 of these 38 donors became non-reactive in subsequent screening (63.16%; 95% CI: 47.28–76.62%). This finding suggests that low S/CO reactivity may often represent transient or non-specific serological reactivity rather than true infection.

### 3.4. Exploratory Analysis of Epidemiological Factors Associated with Confirmed HTLV Infection

To explore potential epidemiological factors associated with HTLV infection, questionnaire data were analyzed for 9 confirmed-positive donors, 6 donors with indeterminate confirmatory results, and 51 confirmed-negative donors. None of the 9 confirmed-positive donors reported travel history to HTLV-endemic countries. In comparison, 1 of 6 indeterminate donors (16.7%) and 12 of 51 confirmed-negative donors (23.5%) reported travel to an HTLV-endemic country, such as Japan. No significant association was observed between travel history to HTLV-endemic countries and confirmed HTLV infection (Table 4).

None of the surveyed participants reported a history of drug use. A history of invasive medical procedures, including surgery, tattooing, or dental procedures, was reported by 8 of 9 confirmed-positive donors (88.9%), 6 of 6 indeterminate donors (100.0%), and 38 of 51 confirmed-negative donors (74.5%). However, this factor was not significantly associated with HTLV infection. A history of multiple sexual partners was reported by 4 of 9 confirmed-positive donors (44.4%), 4 of 6 indeterminate donors (66.7%), and 8 of 18 confirmed-negative donors with available data (44.4%), with no statistically significant difference between groups (Table 4).

A Fujian-related background was reported for 6 of the 9 confirmed-positive donors (66.7%), compared with 23 of 51 confirmed-negative donors (45.1%). The 6 positive donors comprised 3 with Fujian origin who had undergone invasive medical procedures in Fujian (one surgery and two ear piercings) and 3 who had previously visited Fujian. When analyzed separately, invasive medical procedures occurring in Fujian were reported by 3 of 9 confirmed-positive donors (33.3%) but by 0 of 51 confirmed-negative donors (0.0%), and this factor remained significantly associated with HTLV infection after Bonferroni adjustment (adjusted *p* = 0.010). Fujian origin/residence was reported by 3 of 9 confirmed-positive donors (33.3%) and 2 of 51 confirmed-negative donors (3.9%), but this association was no longer significant after adjustment (adjusted *p* = 0.083). Post hoc power estimates are provided in Table 4. Power to detect the observed associations was low for the non-significant comparisons (0.155 for Fujian-related background, OR = 2.25; 0.046 for invasive medical procedures, OR = 1.99; 0.038 for multiple sexual partners, OR = 1.01), illustrating the limited ability of this small exploratory sample to detect modest associations. The detailed questionnaire responses and epidemiological characteristics of each confirmed-positive donor are provided in Table S3.

### 3.5. Familial Testing Among Relatives of Confirmed-Positive Donors

Because sexual transmission and mother-to-child transmission are recognized routes of HTLV transmission, family members of confirmed-positive donors were invited for HTLV testing (Figure 5). A total of 10 relatives from 7 confirmed-positive donors were tested. Among them, two of the 10 tested relatives were HTLV-positive (20.0%; 95% CI: 5.67–50.98%). At the family level, 2 of the 7 families had at least one infected relative, corresponding to a family-level clustering proportion of 28.6%.

The two infected relatives represented different family relationships with the index donors. One confirmed-positive case was the younger sister of donor T5, suggesting the possibility of shared maternal exposure, early-life household exposure, or other common-source intrafamilial transmission. However, because the parents were unavailable for testing, the exact transmission route could not be determined. The other confirmed-positive case was the wife of donor T12. Sexual transmission is plausible, but it cannot be demonstrated without molecular epidemiological evidence. The remaining 8 tested relatives were HTLV negative. The detection of HTLV infection among relatives supports the potential value of family counseling and voluntary testing for close relatives of confirmed-positive blood donors.

## 4. Discussion

This decade-long retrospective study provides an epidemiological characterization of HTLV infection among blood donors in Shenzhen, a large and highly mobile metropolitan city in southern China. Among 512,200 screened donors, 203 were repeatedly reactive by initial ELISA screening, whereas only 18 were confirmed-positive and 10 yielded indeterminate confirmatory results. These findings indicate that HTLV infection in Shenzhen blood donors is uncommon but remains detectable. The low confirmed-positive rate is consistent with the overall epidemiological profile of HTLV infection in China, where national prevalence is low but marked geographic heterogeneity exists, with Fujian Province consistently identified as a relatively higher-prevalence region (Table S4 and Figure S1) [19,21,22,23,24,25,26,27,28,29]. While the screening-reactive rate declined significantly over the study period, the confirmed-positive rate remained low and showed no significant declining trend. This decline persisted after excluding the incomplete 2013 data (RR/year = 0.921, 95% CI: 0.870–0.974, *p* = 0.004), whereas the confirmed-positive rate still showed no significant trend (RR/year = 0.941, 95% CI: 0.777–1.134, *p* = 0.523). However, the trend analyses could not fully account for changes in donor composition (age, sex, first-time versus repeat donation status, and provincial origin), donor-deferral policies, or the different screening assays and confirmatory algorithms used over time. The observed decline may therefore partly reflect assay or time-period effects rather than true epidemiological changes, and interpreting it as evidence of improved screening practices would be conjectural without data controlling for these factors. To compare the prevalence observed in Shenzhen with that reported in other regions of China, published studies reporting HTLV seroprevalence among blood donors in China, together with the present study, are summarized in Table S4 [19,21,22,23,24,25,26,27,28,29], and the geographical distribution of confirmed prevalence estimates is illustrated in Figure S1.

In this low-prevalence donor population, the PPV of initial ELISA reactivity was limited. Of the 203 initially reactive donors, only 18 were confirmed-positive, indicating that most initially reactive results represented false-positive, non-specific, or unconfirmed serological reactivity. This pattern is expected in low-prevalence settings, where even screening assays with acceptable analytical performance generate a substantial proportion of false-positive results. Therefore, confirmatory testing remains indispensable for accurate classification of HTLV infection, appropriate donor deferral, and post-donation counseling. The present findings support the principle that ELISA reactivity should be interpreted as a preliminary screening signal rather than definitive evidence of infection.

The S/CO value appears to provide a useful indicator for predicting confirmatory outcomes among screening-reactive donors. Confirmed-positive samples generally showed high S/CO values, whereas low-level reactivity, particularly values close to the cutoff, often became non-reactive during longitudinal follow-up. Thus, S/CO-based stratification may help optimize confirmatory testing priorities, follow-up intervals, and donor counseling in low-prevalence screening programs. Nevertheless, S/CO values should not be used as a substitute for confirmatory assays, as the S/CO value is only a relative screening signal and may vary according to reagent platform, laboratory conditions, and population prevalence. Like low-level reactivity, indeterminate results also required longitudinal follow-up to clarify their significance. Among the 10 donors with indeterminate confirmatory results, three became non-reactive during follow-up, while the remaining seven remained indeterminate. Moreover, because we obtained only the final interpretation results and did not have access to the original immunoblot strips, we could not retrospectively determine whether any of these indeterminate results represented HGIP-type. In a nationwide survey of Chinese blood donors, seven followed indeterminate donors showed divergent outcomes—one became negative, four remained indeterminate, and two seroconverted at 85 and 250 days after the last donation [19]. Although no seroconversion was observed among our followed indeterminate donors, indeterminate results may occasionally represent early infection, and continued follow-up and deferral of these donors remain necessary to ensure blood safety.

HTLV is transmitted primarily through mother-to-child contact, sexual contact, blood transfusion and invasive medical procedures. In this study, factors related to these transmission routes were analyzed using questionnaire data. Six of the nine confirmed-positive donors who completed the questionnaire reported originating from or residing in Fujian, having previously been to Fujian, or having undergone medical procedures in Fujian. Among these factors, only invasive medical procedures occurring in Fujian remained significantly associated with HTLV infection after Bonferroni adjustment (adjusted *p* = 0.010). However, this association should be interpreted conservatively because this category encompassed heterogeneous types of invasive procedures (e.g., surgery and ear piercing); this association is therefore regarded as hypothesis-generating rather than as evidence of an established epidemiological risk factor. Several limitations of the questionnaire analyses should be acknowledged. The number of confirmed-positive donors was small, which limited statistical power for risk-factor analysis, and only half of the confirmed-positive donors completed the questionnaire, introducing potential selection bias. With only nine cases, meaningful multivariable adjustment is not feasible, and even univariable ORs are highly unstable. Moreover, the wide 95% CI substantially limits the practical value of these estimates; the observed associations should therefore be regarded as exploratory and require confirmation in larger cohorts or multicenter studies with more confirmed-positive cases. In addition, exposure histories were self-reported and may have been affected by recall bias or social-desirability bias, and potential individual-level confounding by age, sex, and geographic origin could not be adequately controlled.

Family testing further indicated possible familial clustering: 2 of 10 tested relatives of 7 confirmed-positive donors were confirmed-positive, including one sibling and one spouse. These findings are compatible with the established transmission routes of HTLV mentioned above. However, parental testing and viral genomic sequencing were not performed. Consequently, we could not determine the direction and timing of transmission within these families or assess the molecular subtype and genetic relatedness of the identified infections. In addition, the tested relatives represented a small, selected subset of all relatives of confirmed-positive donors; the observed proportion (2 of 10) should therefore not be interpreted as an estimate of HTLV prevalence among relatives of infected donors in general.

Finally, because blood donors represent a healthy, pre-screened population aged 18–65 years, these findings cannot be directly generalized to the general population or to higher-risk groups. In addition, individuals with symptomatic HTLV-associated disease are unlikely to be eligible for or present for blood donation, creating additional selection toward a healthier donor population.

## 5. Conclusions

In conclusion, this study demonstrated an extremely low confirmed prevalence of HTLV among Shenzhen blood donors over a ten-year period, accompanied by a significant decline in screening reactivity. Initial screening S/CO values were strongly associated with confirmatory results. Fujian-related invasive medical procedures were more common among the small subset of surveyed HTLV-positive donors. Two positive relatives were identified among 10 relatives tested, suggesting possible familial clustering; however, the direction and route of transmission could not be established. Both findings are exploratory and require confirmation in larger studies with adequate statistical power.

## Figures and Tables

**Figure 1:**
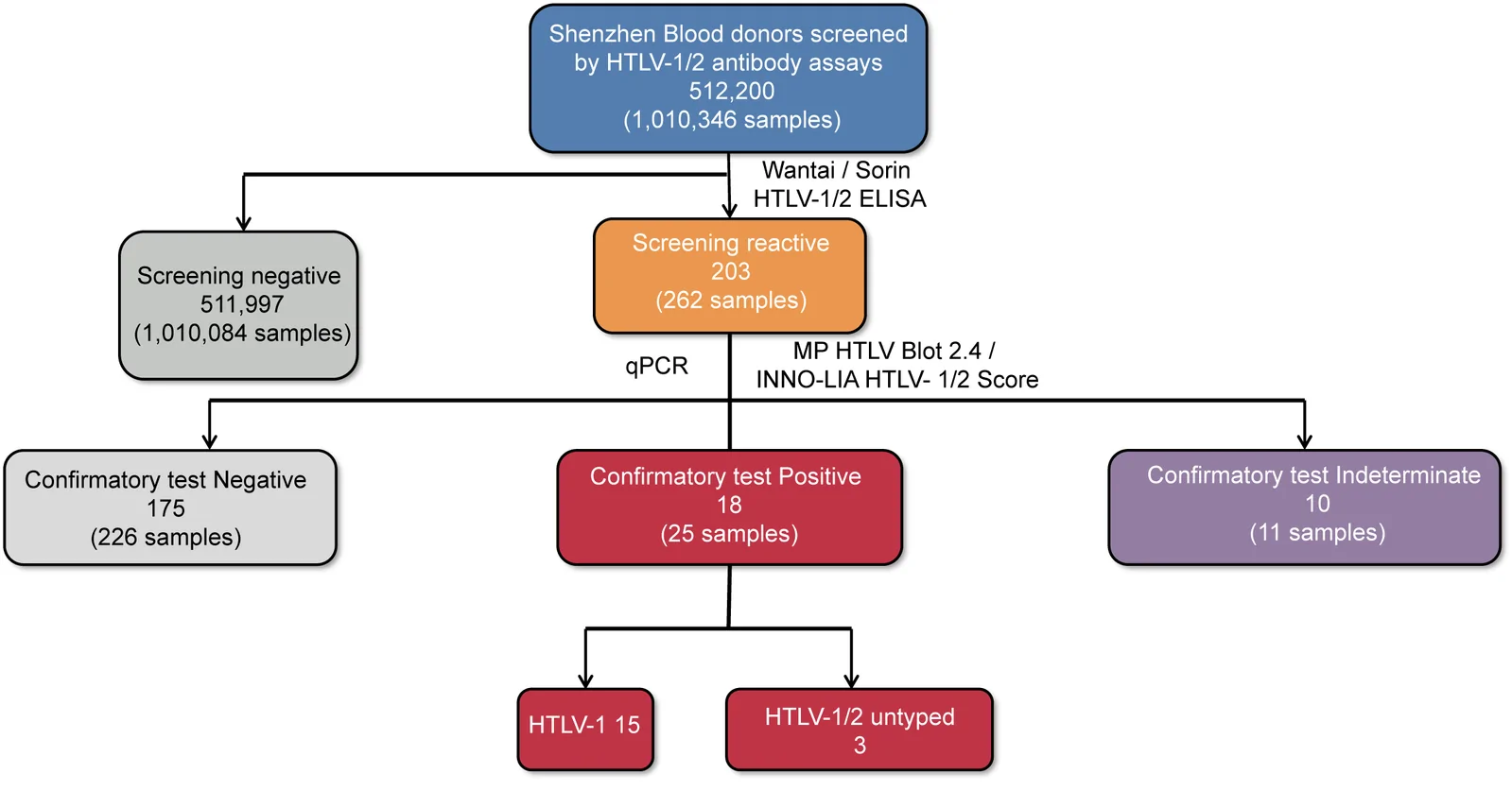
Screening and confirmation process for Human T-lymphotropic virus (HTLV)-1/2 among blood donors at Shenzhen Blood Center. One donor may contribute multiple samples. The three HTLV-1/2 untyped donors were confirmed with the MP HTLV Blot 2.4 assay, for which typing results were not documented.

**Figure 2:**
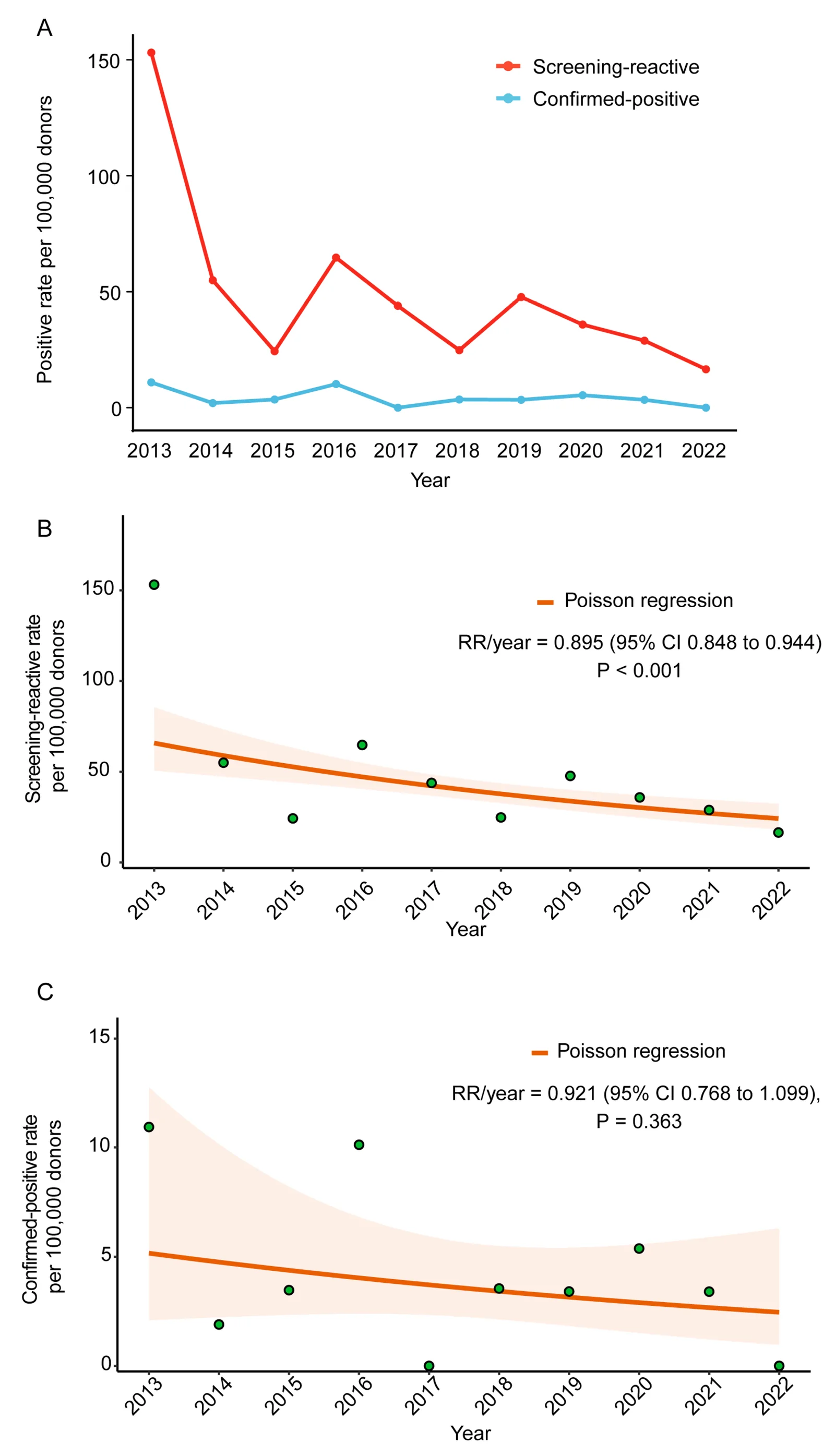
Changes in HTLV screening-reactive and confirmed-positive rates among blood donors in Shenzhen over a 10-year period. (**A**) Annual screening-reactive and confirmed-positive rates per 100,000 donors from 2013 to 2022. The upper red curve represents the screening-reactive rate, and the lower blue curve represents the confirmed-positive rate. (**B**) Poisson regression analysis of screening-reactive counts over time. (**C**) Poisson regression analysis of confirmed-positive counts over time.

**Figure 3:**
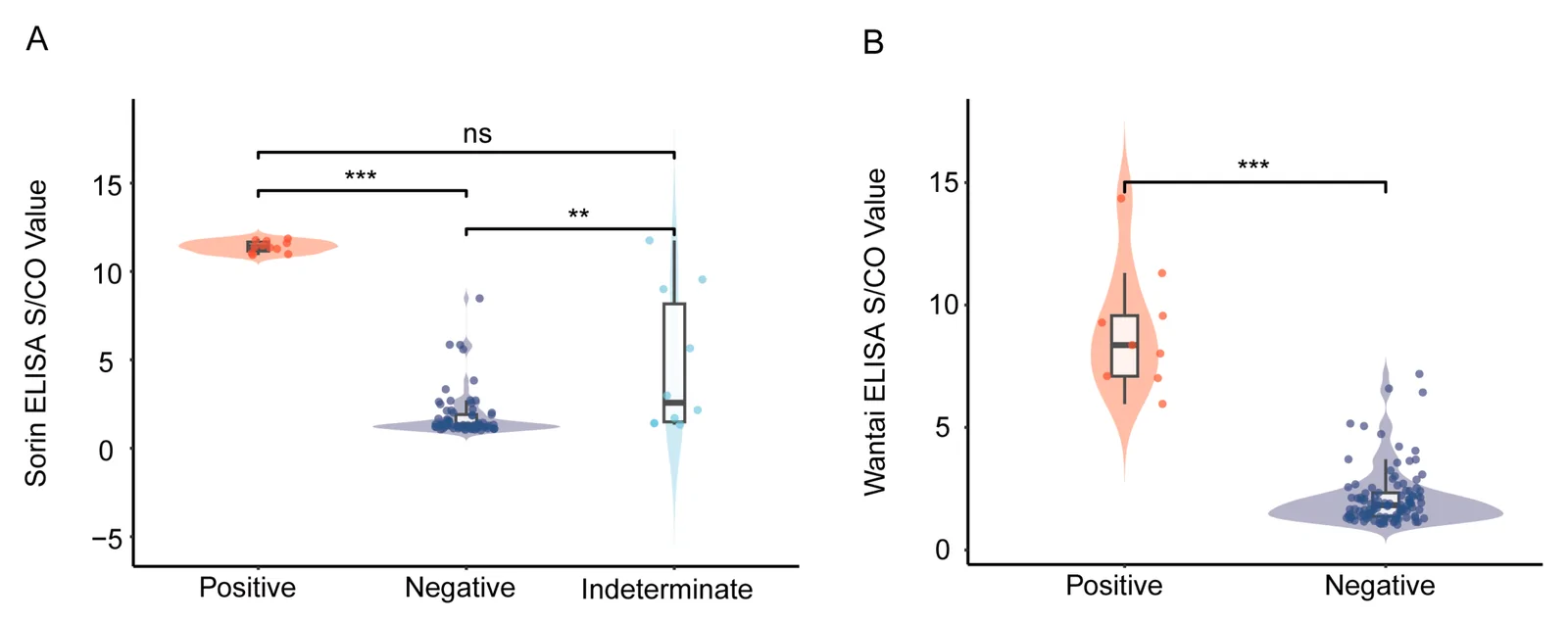
Comparison of initial screening S/CO values among blood donors with different confirmatory outcomes. For donors with repeated donations, S/CO measurements were averaged within each assay, so that each donor contributed one value per assay. (**A**) Comparison of Sorin S/CO values among the confirmed-positive, confirmed-negative, and indeterminate groups. The overall comparison was performed using the Kruskal–Wallis test, followed by Dunn’s post hoc tests with Holm adjustment. (**B**) Comparison of Wantai S/CO values between the confirmed-positive and confirmed-negative groups using a two-sided Mann–Whitney U test. (ns, *p* ≥ 0.05; **, *p* < 0.01; ***, *p* < 0.001).

**Figure 4:**
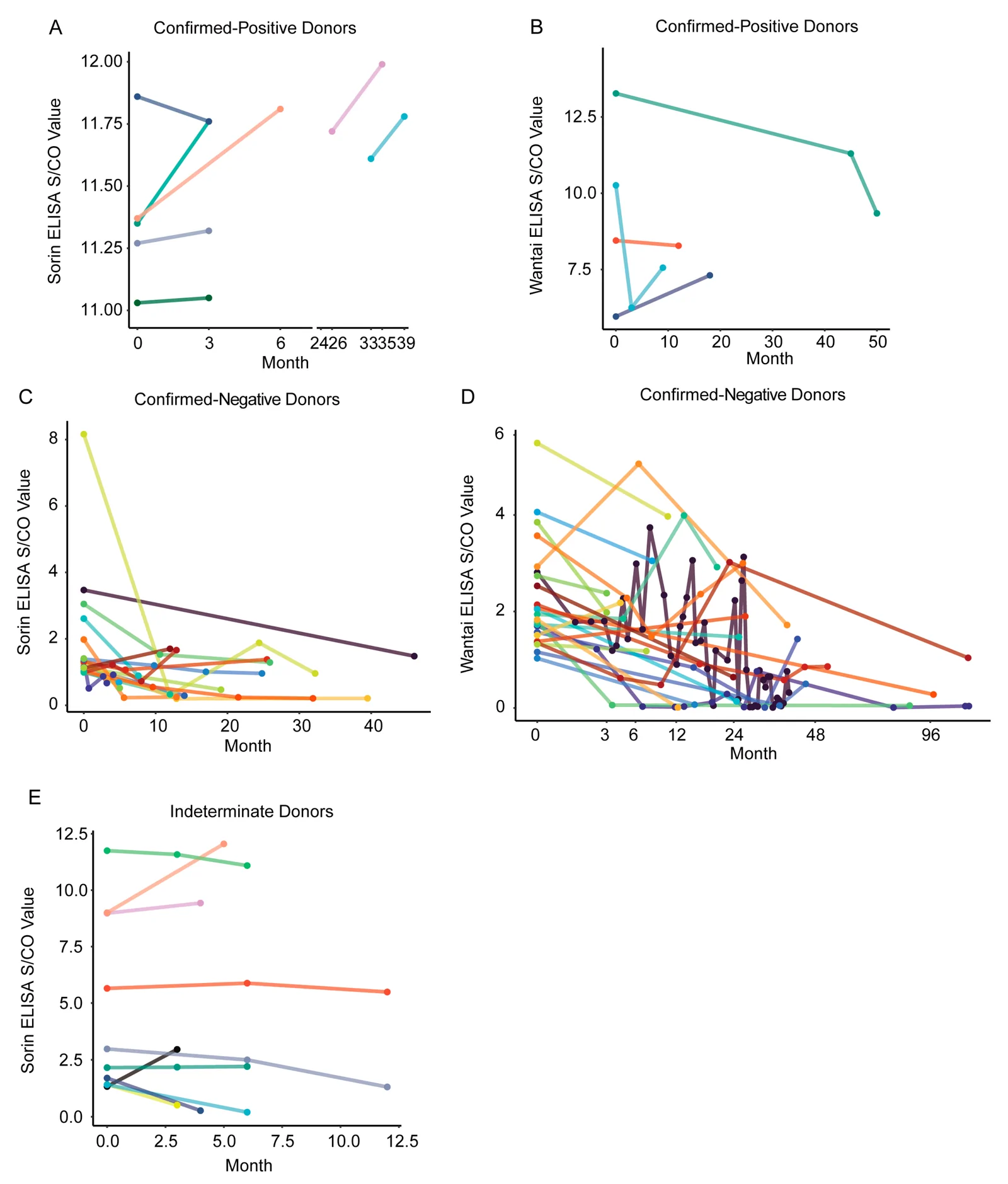
Long-term follow-up of HTLV screening-reactive blood donors. (**A**) Dynamic changes in S/CO values of screening-reactive results using the Sorin assay in confirmed-positive blood donors (*n* = 7); (**B**) dynamic changes in S/CO values of screening-reactive results using the Wantai assay in confirmed-positive blood donors (*n* = 4); (**C**) dynamic changes in S/CO values of screening-reactive results using the Sorin assay in confirmed-negative blood donors (*n* = 18); (**D**) dynamic changes in S/CO values of screening-reactive results using the Wantai assay in confirmed-negative blood donors (*n* = 23); (**E**) dynamic changes in S/CO values of screening-reactive results using the Sorin assay in blood donors with indeterminate confirmatory results (*n* = 10).

**Figure 5:**
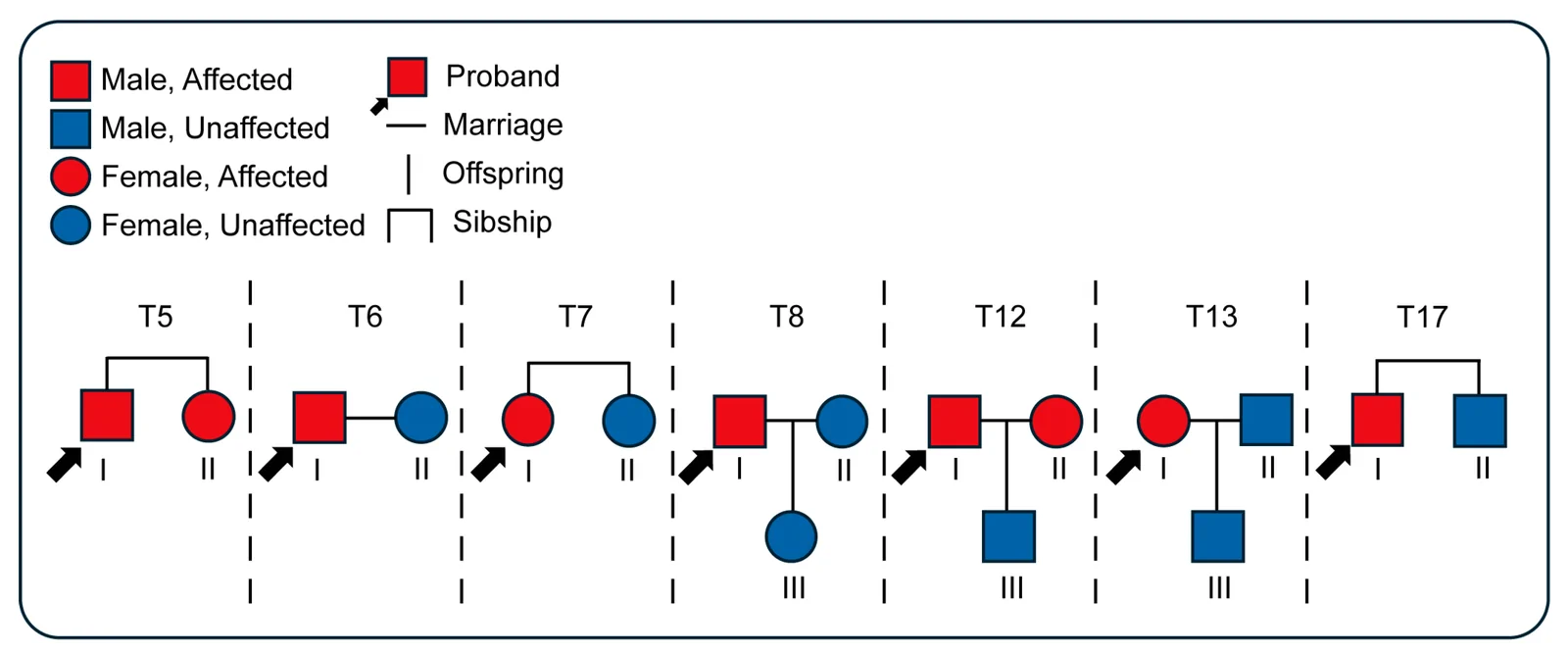
Pedigree charts of HTLV infection among relatives of confirmed-positive donors. Detailed definitions of symbols and patterns are provided in the legend at the upper left.

**Table 1:** Annual HTLV screening and confirmatory outcomes among blood donors in Shenzhen, 2013–2022.

Year	Number of Donors Screened	Screening-Reactive Donors	Screening-Reactive Rate per 100,000 (95% CI)	Confirmed-Positive Donors	Confirmed-Positive Rate per 100,000 (95% CI)	Indeterminate Donors	Initial Screening PPV
2013	9142	14	153.14 (83.72–256.94)	1	10.94 (0.28–60.95)	/	7.14%
2014	52,721	29	55.01 (36.84–79.00)	1	1.90 (0.05–10.57)	/	3.45%
2015	57,619	14	24.30 (13.28–40.77)	2	3.47 (0.42–12.54)	/	14.29%
2016	49,401	32	64.78 (44.31–91.44)	5	10.12 (3.29–23.62)	/	15.63%
2017	59,287	26	43.85 (28.65–64.26)	0	0.00 (/)	/	0.00%
2018	56,371	14	24.84 (13.58–41.67)	2	3.55 (0.43–12.82)	3	14.29%
2019	58,666	28	47.73 (31.71–68.98)	2	3.41 (0.41–12.31)	4	7.14%
2020	55,765	20	35.86 (21.91–55.39)	3	5.38 (1.11–15.72)	2	15.00%
2021	58,766	17	28.93 (16.85–46.32)	2	3.40 (0.41–12.29)	1	11.76%
2022	54,462	9	16.53 (7.56–31.37)	0	0.00 (/)	0	0.00%
Total	512,200	203	39.63 (34.37–45.48)	18	3.51 (2.08–5.55)	10	8.87%

Note: For 2013, only donors who donated between 26 February and 22 April were included. All numbers in this table refer to donors, as distinct from samples.

**Table 2:** Confirmatory outcomes of screening-reactive samples stratified by signal-to-cutoff (S/CO) value.

Reagent	S/CO Category	Number of Donors	Confirmed-Positive	Indeterminate	Confirmed-Negative	Confirmed-Positive Proportion
Sorin	1–2	57	0	4	53	0
	>2–5	14	0	2	12	0
	>5–10	7	0	3	4	0
	>10	12	11	1	0	91.67%
Wantai	1–2	64	0	0	64	0
	>2–5	38	0	0	38	0
	>5–10	12	7	0	5	58.33%
	>10	2	2	0	0	100%

**Table 3:** Longitudinal follow-up trajectories of initially reactive donors.

Initial Serological Profile	Number of Donors	Persistently Reactive and Confirmed-Positive	Persistently Reactive and Indeterminate	Became Non-Reactive During Follow-Up	Remained Screening-Reactive but Confirmed-Negative
Confirmed-positive	11	11	0	0	0
Indeterminate confirmatory result	10	0	7	3	0
Confirmed-negative	41	0	0	26	15

**Table 4:** Questionnaire-based epidemiological factors associated with confirmed HTLV infection.

Risk Factor	Confirmed-Positive *n*/N (%)	Confirmed-Negative *n*/N (%)	OR	95% CI	*p* Value	Adjusted *p* Value	Post Hoc Power
Travel to HTLV-endemic countries	0/9 (0.0%)	12/51 (23.5%)	/	/	/	/	/
Invasive medical procedures	8/9 (88.9%)	38/51 (74.5%)	1.99	0.31–12.55	0.608	/	0.046
Multiple sexual partners	4/9 (44.4%)	8/18 (44.4%)	1.01	0.22–4.71	1.000	/	0.038
Fujian-related background	6/9 (66.7%)	23/51 (45.1%)	2.25	0.55–9.22	0.292	/	0.155
Fujian origin/residence	3/9 (33.3%)	2/51 (3.9%)	10.66	1.73–65.63	0.021 *	0.083	0.615
Invasive medical procedures occurring in Fujian	3/9 (33.3%)	0/51 (0%)	55.46	2.57–1198.94	0.002 **	0.01 *	0.857

Note: Of the 18 confirmed-positive donors, 9 completed the questionnaire. The 51 control questionnaires were voluntarily completed by blood donors recruited during a one-month period without matching or frequency matching on age, sex, year of donation, or other variables. For the multiple sexual partners item, responses were available for only 18 of the 51 controls (33 questionnaires missing). OR and 95% CI were calculated using the Haldane–Anscombe correction. Fisher’s exact test was used for the Fujian subgroup comparisons, and continuity-corrected chi-square tests were used for the remaining comparisons. Bonferroni-adjusted *p* values are reported for the Fujian-related comparisons. (* *p* < 0.05; ** *p* < 0.01).

## Data Availability

All data supporting the findings of this study are available within this paper and the Supplementary Materials.

## Supplementary Materials

The following supporting information can be downloaded at: https://www.mdpi.com/article/10.3390/v18101058/s1: File S1: Questionnaire on conditions related to Human T-lymphotropic virus (HTLV) infection; Table S1: Follow-up test results of confirmed HTLV-positive blood donors; Table S2: Follow-up test results of indeterminate blood donors; Table S3: Epidemiological characteristics and follow-up information of confirmed HTLV-positive blood donors in Shenzhen; Table S4: Summary of studies reporting HTLV seroprevalence among blood donors in China, including the present study; Figure S1: Geographical distribution of confirmed HTLV seroprevalence among blood donors in China.

## Abbreviations

The following abbreviations are used in this manuscript:

HTLV	Human T-lymphotropic virus
SZBC	Shenzhen Blood Center
ELISA	Enzyme-linked immunosorbent assay
NCCL	National Center for Clinical Laboratories
PPV	Positive predictive value
OR	Odds ratio
CI	Confidence interval
RR	Rate ratio
PVL	Proviral load
S/CO	Signal-to-cutoff

## References

[1] Poiesz B.J., Ruscetti F.W., Gazdar A.F., Bunn P.A., Minna J.D., Gallo R.C. (1980). Detection and isolation of type C retrovirus particles from fresh and cultured lymphocytes of a patient with cutaneous T-cell lymphoma. Proc. Natl. Acad. Sci. USA.

[2] Proietti F.A., Carneiro-Proietti A.B., Catalan-Soares B.C., Murphy E.L. (2005). Global epidemiology of HTLV-I infection and associated diseases. Oncogene.

[3] Kalyanaraman V.S., Sarngadharan M.G., Robert-Guroff M., Miyoshi I., Golde D., Gallo R.C. (1982). A new subtype of human T-cell leukemia virus (HTLV-II) associated with a T-cell variant of hairy cell leukemia. Science.

[4] Martinez M.P., Al-Saleem J., Green P.L. (2019). Comparative virology of HTLV-1 and HTLV-2. Retrovirology.

[5] Roucoux D.F., Murphy E.L. (2004). The epidemiology and disease outcomes of human T-lymphotropic virus type II. AIDS Rev..

[6] Calattini S., Chevalier S.A., Duprez R., Bassot S., Froment A., Mahieux R., Gessain A. (2005). Discovery of a new human T-cell lymphotropic virus (HTLV-3) in Central Africa. Retrovirology.

[7] Wolfe N.D., Heneine W., Carr J.K., Garcia A.D., Shanmugam V., Tamoufe U., Torimiro J.N., Prosser A.T., Lebreton M., Mpoudi-Ngole E. (2005). Emergence of unique primate T-lymphotropic viruses among central African bushmeat hunters. Proc. Natl. Acad. Sci. USA.

[8] Willems L., Hasegawa H., Accolla R., Bangham C., Bazarbachi A., Bertazzoni U., Carneiro-Proietti A.B., Cheng H., Chieco-Bianchi L., Ciminale V. (2017). Reducing the global burden of HTLV-1 infection: An agenda for research and action. Antivir. Res..

[9] Vrielink H., Reesink H.W. (2004). HTLV-I/II prevalence in different geographic locations. Transfus. Med. Rev..

[10] Murphy E.L. (2016). Infection with human T-lymphotropic virus types-1 and -2 (HTLV-1 and -2): Implications for blood transfusion safety. Transfus. Clin. Biol. J. Soc. Fr. Transfus. Sang..

[11] Satake M., Yamaguchi K., Tadokoro K. (2012). Current prevalence of HTLV-1 in Japan as determined by screening of blood donors. J. Med. Virol..

[12] Hindawi S., Badawi M., Fouda F., Mallah B., Mallah B., Rajab H., Madani T.A. (2018). Testing for HTLV 1 and HTLV 2 among blood donors in Western Saudi Arabia: Prevalence and cost considerations. Transfus. Med..

[13] da Silva Brito V., Santos F.L.N., Gonçalves N.L.S., Araujo T.H.A., Nascimento D.S.V., Pereira F.M., Boa-Sorte N.C.A., Grassi M.F.R., Caterino-de-Araujo A., Galvão-Castro B. (2018). Performance of commercially available serological screening tests for Human T-Cell Lymphotropic Virus infection in Brazil. J. Clin. Microbiol..

[14] Reynolds C., Davison K.L., Brailsford S.R. (2019). Safe supplies: Few infections in UK blood and tissue donors. Transfus. Med..

[15] Inaba S., Sato H., Okochi K., Fukada K., Takakura F., Tokunaga K., Kiyokawa H., Maeda Y. (1989). Prevention of transmission of human T-lymphotropic virus type 1 (HTLV-1) through transfusion, by donor screening with antibody to the virus. One-year experience. Transfusion.

[16] Laperche S., Worms B., Pillonel J. (2009). Blood safety strategies for human T-cell lymphotropic virus in Europe. Vox Sang..

[17] Gessain A., Ramassamy J.L., Afonso P.V., Cassar O. (2023). Geographic distribution, clinical epidemiology and genetic diversity of the human oncogenic retrovirus HTLV-1 in Africa, the world’s largest endemic area. Front. Immunol..

[18] Twizere J.C., Rosadas C., Kidiga M., Djalo F., Murphy E.L., Mouinga-Ondeme A., Vermeulen M., Morrell L., Diop S., Maseko S. (2025). Strengthening awareness and response to HTLV-1 infection in Africa: A neglected threat to blood safety and public health. HemaSphere.

[19] Chang L., Ou S., Shan Z., Zhu F., Ji H., Rong X., Guo F., Jiang X., Sun H., Yan Y. (2021). Seroprevalence of human T-lymphotropic virus infection among blood donors in China: A first nationwide survey. Retrovirology.

[20] Ji H., Chang L., Yan Y., Wang L. (2023). Development and validation of a duplex real-time PCR for the rapid detection and quantitation of HTLV-1. Virol. J..

[21] Ji H., Chang L., Yan Y., Sun H., Liu Y., Wang L. (2023). Genetic typing and intrafamilial transmission of human T-lymphotropic virus type 1 in non-endemic areas of China. Front. Microbiol..

[22] Zhao J., Zhao F., Han W., Xu X., Wang L., Li R., Li T., Wu L., Du D., Zeng X. (2020). HTLV screening of blood donors using chemiluminescence immunoassay in three major provincial blood centers of China. BMC Infect. Dis..

[23] Liao Q., Shan Z., Wang M., Huang J., Xu R., Li T., Wang W., Li C., Rong X., Fu Y. (2021). Prevalence and evolutionary analyses of human T-cell lymphotropic virus in Guangdong province, China: Transcontinental and Japanese subtype lineages dominate the prevalence. PLoS Neglected Trop. Dis..

[24] Wang Y., Li X., Song A., Zhang C., Chen Y., Chen C., Lin Y., Shun L., Li L., Liu Y. (2005). Prevalence and partial sequence analysis of human T cell lymphotropic virus type I in China. J. Med. Virol..

[25] Li L., Ou S., Huang C., Zhou X., Ge H., Li J., Zeng J., Zhou A., He L., Xu Q. (2019). The prevalence of human T-cell leukemia virus in blood donors in China. Transfusion.

[26] Chen X., Liu F., Fu X., Feng Y., Zhang D., Liu H., Xi G., Hu J. (2019). Prevalence of human T-cell lymphotropic virus type-1 infection among blood donors in mainland China: A systematic review and meta-analysis of the last 20 years. Expert Rev. Hematol..

[27] Xie J., Ge S., Zhang Y., Lin Y., Ni H., Zhang J., Chen C. (2015). The prevalence of human T-lymphotropic virus infection among blood donors in southeast China, 2004–2013. PLoS Neglected Trop. Dis..

[28] Mengjiao L., Yushan X., Yan L., Dawei C., Xiaojun Z., Yongjun W., Cuifen S., Jue X. (2024). Prevalence of transfusion-transmitted infections in hospitalized patients before transfusion and volunteer blood donors in Zhejiang Province, China. Infect. Dis. Now.

[29] Lee Y.H., Lu C.T., Feng I.J., Hung C.M. (2023). Trend in seroprevalence of HTLV-1/2 in Taiwanese blood donors-A 10-year follow-up. Transfus. Med..

