# Supplementary material for: HTLV Seroprevalence and Associated Factors Among Blood Donors in Shenzhen, China: A Cross-Sectional Study with Longitudinal Follow-Up, 2013–2022
Source: Viruses. 2026 Sep 24;18(10):1058. doi: 10.3390/v18101058 (PMC13709397; doi:10.3390/v18101058)
Supplement: Supplementary file 1 [file viruses-18-01058-s001.zip › File S1 Questionnaire on conditions related to Human T-lymphotropic virus (HTLV) infection.pdf]

Questionnaire on conditions related to Human T-lymphotropic virus (HTLV) infection

ID Number:________________/________________

Notes:__________________________________

Hello! I am an employee from _____________. I am conducting a survey on HTLV infections. We would like to know about your basic situation and health condition. Your answers to any of the following questions are confidential and your name will not appear on the survey. If you have any questions, please feel free to ask. Your honest answers to these questions will help us better understand the status of HTLV infection in our country. Thank you for your cooperation!

Instructions for completion: For multiple choice questions (single/multiple choice), please check the box directly on the option. Fill in the "_______" accordingly.

Part I. Basic information

1. Your birth sex:

   A. Male B. Female

2. Your date of birth:_______Year________Month_______Date

3. Your place of origin:_______Province______City, Birthplace:_______Province______City, Growing place:______Province______City

4. Your ethnicity:____________

5. Your level of education:

   A. Unschooled B. Secondary schools C. Junior high school D. High school

   E. Junior college F. Undergraduate G. Master's degree or Ph.D. Or higher

6. Your marital status:

   A. Unmarried B. Married C. Cohabitation D. Divorced or widowed

7. Your occupation:

   A. Student B. Worker C. Company employee D. Medical staff

E. Farmer F. Soldier G. Civil servant H. Individual operation I. Teacher J. Freelancer

K. Unemployed L. Other (please indicate) ____________________

8. Your current family residence:

A. Village B. County/town C. Small-medium city D. Big city (Municipality、provincial capital)

Part II. Work and life situation

9. How long have you lived here? _________Year________Month

(*Enter the number of months if less than 1 year, or 1 month if less than 1 month)*

10. Have you ever changed your residence *(Cross-city)*?

A. Yes (_______number of times, City:_______________) B. No

11. Your current monthly income (RMB):

A. No income. Use your own or your family's savings, pension, etc.

B. 1～999 C. 1000～1999 D. 2000～4999

E. 5000～9999 F. 10000～19999 G. ≥20000

12. Your current monthly household income (RMB):

A. No income B. 1～999 C. 1000～1999 D. 2000～4999

E. 5000～9999 F. 10000～19999 G. ≥20000

13. The number of children you currently have is:

A. 0 (Go to question 15) B. 1 C. 2 D. 3 E. More than 3

14. Is your child breastfed (*If yes, please indicate the duration of breastfeeding, if more than one child, please indicate the duration separately)*:

A. Yes, Breastfeeding for _______________ month (Child 1)________month (Child 2) month (Child 3) B. No

15. Have you visited the following areas of the country for work, study or travel, etc. (Multiple choices allowed) (If yes, please indicate the duration of your stay):

A. Zhejiang Province______ B. Fujian Province_______

C. Guangdong province_______ D. No

16. Have you visited the following areas abroad for work, study or travel, etc. (multiple choice) (if

yes, please indicate the duration of your stay):

A. Japan______ B. Iran______ C. Caribbean (e.g., Jamaica)______

D. Colombia______ E. Brazil______ F. Sub-Saharan Africa______ G. No

Part III. Medical conditions

17. Have you experienced any of the following? (*In the space for questions a~k, fill in the corresponding answer serial number, respectively. If yes, please give details of the number of times, time and place.)*

A. Yes B. No C. Unknown

A|__| Extractions, cleaning, filling or fixed teeth	Number of times:	Time:	Place:
B|__| Tattoo	Number of times:	Time:	Place:
C|__| Ear piercing	Number of times:	Time:	Place:
D|__| Shared toothbrush	Number of times:	Time:	Place:
E|__| Acupuncture	Number of times:	Time:	Place:
F|__| Donate blood	Number of times:	Time:	Place:
G|__| Receiving blood transfusions or other blood products	Number of times:	Time:	Place:
H|__| Surgeries	Number of times:	Time:	Place:
I|__| Suture treatment (excluding surgery)	Number of times:	Time:	Place:
J|__| Puncture therapy or interventional therapy	Number of times:	Time:	Place:
K|__| Endoscopy (gastroscopy, enteroscopy or colposcopy)	Number of times:	Time:	Place:

18. Have you ever been diagnosed with any of the following (multiple choice):

A. Adult T cell leukemia B. HTLV-associated myelopathy/tropical spastic paralysis

C. Uveitis D. Chronic inflammatory arthritis E. Infectious dermatitis

F. Polymyositis G. Bronchopulmonary disease H. Herpes labialis I. No

Part IV. Behavioral situation

19.1. Have you ever injected drugs (if yes, please indicate the number of times):

A. Yes (____number of times) B. No C. Refuse to answer

19.2. Have you ever shared needles (if yes, please indicate the number of times):

A. Yes (____number of times) B. No C. Refuse to answer

20.1. Have you ever had sexual intercourse:

A. Yes B. No *(Closing the Questionnaire)* C. Refuse to answer *(Closing the Questionnaire)*

20.2. The age you had your first sexual intercourse was ______ years old.

20.3. Total of ______ person you have had sexual relations with so far.

20.4. Do you use condoms when having sex?

A. Every time B. Frequent C. Infrequent D. Never

Answers 21.1-21.3 for those whose marital status is married/cohabiting/divorced or widowed

21.1. To date, you have had ______ marriage/cohabitation, the longest of which lasted.

______ years and months.

21.2. How often you have sex with him/her: ______times/month.

21.3. How often did you use a condom when you had sex with him/her?

A. Every time B. Frequent C. Infrequent D. Never

22.1. Have you ever had sex with a commercial sex partner (if yes, please indicate the number of times)

A. Yes, _____number of times B. No *(Closing the Questionnaire)*

C. Refuse to answer *(Closing the Questionnaire)*

22.2. How often have you had sex with a commercial sex partner using a condom:

A. Every time B. Frequent C. Infrequent D. Never

End of questionnaire, thank you very much for your cooperation!

Signature of investigator: ______________________

Dates: _________Year ______Month _______Day

Signature of auditor: ______________________

Dates: _________Year ______Month _______Day
