# Supplementary material for: HTLV Seroprevalence and Associated Factors Among Blood Donors in Shenzhen, China: A Cross-Sectional Study with Longitudinal Follow-Up, 2013–2022
Source: Viruses. 2026 Sep 24;18(10):1058. doi: 10.3390/v18101058 (PMC13709397; doi:10.3390/v18101058)
Supplement: Supplementary file 1 [file viruses-18-01058-s001.zip › Supplementary Materials.pdf]

Table S1 Follow-up test results of confirmed HTLV-positive blood donors

No.	Detection interval (Months)	ELISA				PCR		WB		
		Reagent	Initial (S/CO)	Repeat(S/CO)	ELISA Result	Measured Value（Ct）	PCR Result	Reagent	WB Result	Final Result
T1	0	WANTAI	12.21	5.66/10.81	Pending Confirmation	/	/	MP	Positive	HTLV-1/2
T2	0	WANTAI	16.21	12.57/14.28	Pending Confirmation	/	/	MP	Positive	HTLV-1/2
T3	0	WANTAI	8.07	4.9/8.07	Pending Confirmation	/	/	MP	Positive	HTLV-1/2
T4	0	WANTAI	6.59	9.11/9.64	Pending Confirmation	26.80/24.93	Positive	MP	Positive	HTLV-1
	12	WANTAI	11.33	6.75/6.75	Pending Confirmation	26.21/27.96	Positive	MP	Positive	HTLV-1
T5	0	WANTAI	7.48	11.54/11.76	Pending Confirmation	28.38/27.62	Positive	MP	Positive	HTLV-1
	3	WANTAI	7.51	5.63/5.61	Pending Confirmation	27.43/26.39	Positive	MP	Positive	HTLV-1
	9	WANTAI	7.81	7.85/7.02	Pending Confirmation	29.96/29.53	Positive	MP	Positive	HTLV-1
	24	Sorin	11.81	11.67/11.67	Pending Confirmation	28.96/27.53	Positive	INNO-LIA	HTLV-1	HTLV-1
T6	0	WANTAI	10	14.97/14.84	Pending Confirmation	/	/	MP	Positive	HTLV-1/2
	45	WANTAI	8.68	13.09/12.13	Pending Confirmation	27.63/27.84	Positive	MP	Positive	HTLV-1

	50	WANTAI	9.24	8.54/10.23	Pending Confirmation	28.53/27.16	Positive	MP	Positive	HTLV-1
T7	0	WANTAI	8.07	4.78/5.03	Pending Confirmation	/	/	MP	Positive	HTLV-1/2
	18	WANTAI	7.65	7.23/7.38	Pending Confirmation	25.7/25.5	Positive	INNO-LIA	HTLV-1	HTLV-1
T8	0	WANTAI	6.98	8.17/6.13	Pending Confirmation	/	/	/	/	/
	26	Sorin	11.81	11.67/11.67	Pending Confirmation	/	/	INNO-LIA	HTLV-1	HTLV-1
	35	Sorin		11.97/12.01	Pending Confirmation	28.80/25.88	Positive	INNO-LIA	HTLV-1	HTLV-1
T9	0	Sorin	11.58	11.86/11.86	Pending Confirmation	36.27	Positive	INNO-LIA	HTLV-1	HTLV-1
T10	0	Sorin	11.21	11.58/11.58	Pending Confirmation	34.5	Positive	INNO-LIA	HTLV-1	HTLV-1
T11	0	WANTAI	9.48	9.07/9.28	Pending Confirmation	32.32/32.98	Positive	MP	Positive	HTLV-1
	33	Sorin	11.58	11.63/11.63	Pending Confirmation	23.55/23.58	Positive	INNO-LIA	HTLV-1	HTLV-1
	39	Sorin		11.78/11.78	Pending Confirmation	23.68/24.01	Positive	INNO-LIA	HTLV-1	HTLV-1
T12	0	Sorin	11.58	11.24/11.24	Pending Confirmation	26.52/26.61	Positive	INNO-LIA	HTLV-1	HTLV-1
	3	/		11.76/11.76	/	5.288(copies/100PBMC)	Positive	INNO-LIA	HTLV-1	HTLV-1

T13	0	Sorin	12.05	11.76/11.76	Pending Confirmation	31.17/30.7	Positive	INNO-LIA	HTLV-1	HTLV-1
	3	/		11.76/11.76	/	0.018(copies/100PBMC)	Positive	INNO-LIA	HTLV-1	HTLV-1
T14	0	Sorin	11.54	11.28/11.28	Pending Confirmation	N/A	Negative	INNO-LIA	HTLV-1	HTLV-1
	6	Sorin	11.81	11.81	Pending Confirmation	N/A	Negative	INNO-LIA	HTLV-1	HTLV-1
T15	0	Sorin	11.41	10.65/10.72	Pending Confirmation	N/A	Negative	INNO-LIA	HTLV-1	HTLV-1
T16	0	Sorin	10.91	11.45/11.45	Pending Confirmation	0.12(copies/100PBMC)	Positive	INNO-LIA	HTLV-1	HTLV-1
	3	Sorin	11.32	11.32	Pending Confirmation	/	/	INNO-LIA	HTLV-1	HTLV-1
T17	0	Sorin	11.03	11.03/11.03	Pending Confirmation	/	/	INNO-LIA	HTLV-1	HTLV-1
	3	Sorin	11.08	11.05/11.01	Pending Confirmation	/	/	/	/	/
T18	0	Sorin	11.46	10.57/10.91	Pending Confirmation	N/A	Negative	INNO-LIA	HTLV-1	HTLV-1

Note: Typing results were not documented for samples tested with the MP assay。

Table S2 Follow-up test results of indeterminate blood donors

No.	Detection interval (months)	ELISA			PCR		WB			Recommendation
		Initial (S/CO)	Repeat(S/CO)	Result	Ct	Result	Reagent	Result	Final result	
Id1	0	1.22	1.53/1.51	ID	N/A	(-)	INNO-LIA	ID	ID	Resample
	3	0.58	0.46/0.5	(-)	N/A	(-)	INNO-LIA	(-)	(-)	Reinstatement of blood donation eligibility
Id2	0	9.4	8.69/8.84	ID	N/A	(-)	INNO-LIA	ID	ID	Resample
	4		9.36/9.49	ID	N/A	(-)	INNO-LIA	ID	ID	Resample
Id3	0	1.36	1.31/1.32	ID	N/A	(-)	INNO-LIA	ID	ID	Resample
	3		2.89/3.02	ID	N/A	(-)	INNO-LIA	ID	ID	Resample
Id4	0	6.89	5.13/4.92	ID	N/A	(-)	INNO-LIA	ID	ID	Resample
	6	5.63	6.02/5.74	ID	N/A	(-)	INNO-LIA	ID	ID	Resample
	12	5.96	5.47/5.51	ID	N/A	(-)	INNO-LIA	ID	ID	Permanent deferral from blood donation
Id5	0	1.3	1.6/1.32	ID	N/A	(-)	INNO-LIA	ID	ID	Resample
	6		0.2/0.2	(-)	N/A	(-)	INNO-LIA	(-)	(-)	Reinstatement of blood donation eligibility

Id6	0	2.67	1.88/1.92	ID	N/A	(-)	INNO-LIA	ID	ID	Resample
	3		2.23/2.13	ID	N/A	(-)	INNO-LIA	ID	ID	Resample
	6		2.16/2.25	ID	N/A	(-)	INNO-LIA	ID	ID	Permanent deferral from blood donation
Id7	0	2.08	1.53/1.5	ID	N/A	(-)	INNO-LIA	ID	ID	Resample
	4		0.27/0.26	(-)	N/A	(-)	INNO-LIA	(-)	(-)	Reinstatement of blood donation eligibility
Id8	0	10.79	8.03/8.18	ID	N/A	(-)	INNO-LIA	ID	ID	Resample
	5		12.05/12.05	ID	N/A	(-)	INNO-LIA	ID	ID	Resample
Id9	0	11.63	11.81/11.81	ID	N/A	(-)	INNO-LIA	ID	ID	Resample
	3		11.58/11.58	ID	N/A	(-)	INNO-LIA	ID	ID	Resample
	6		11.05/11.12	ID	N/A	(-)	INNO-LIA	ID	ID	Permanent deferral from blood donation
Id10	0	3.08	3.15/2.71	ID	N/A	(-)	INNO-LIA	ID	ID	Resample
	6		2.48/2.51	ID	N/A	(-)	INNO-LIA	ID	ID	Resample
	12		1.31/1.3	ID	N/A	(-)	INNO-LIA	ID	ID	Permanent deferral from blood donation

ID: Indeterminate

Table S3 Epidemiological characteristics and follow-up information of confirmed HTLV-positive blood donors in Shenzhen

No.	Age	Gender	Place of origin	High risk behaviors	High risk areas	Relative
T1	32	Female	Guangdong	-	-	-
T2	56	Male	Fujian	-	-	-
T3	42	Female	Hubei	-	-	-
T4	40	Male	Fujian	-	-	-
T5	32	Male	Fujian	Surgery in Fujian in 1991 / Multiple sexual partners	Fujian/ Zhejiang/ Guangdong	Sister(+)
T6	34	Male	Guangdong	-	-	Wife(-)
T7	37	Female	Hunan	Ear piercing 20 years ago/ surgery/ Multiple sexual partners / Dental procedure	Fujian (5-6 days)/Guang dong	Elder sister(-)
T8	32	Male	Guangdong	Tattooing/ Wound suturing	-	Wife(-) Daughter(-)
T9	40	Female	Guangdong	-	-	-
T10	45	Male	Fujian	-	-	-

T11	36	Female	Fujian	Ear piercing in Fujian in 1999/Multiple sexual partners /Acupuncture/Endoscopic procedure/Dental procedure	Fujian/ Guangdong	-
T12	55	Male	Guangdong	Endoscopic procedure in 1996/Dental procedure/Surgery	Guangdong/ Zhejiang (1 day)	Wife(+) Son(-)
T13	53	Female	Hubei	Sutural therapy in a small hospital in 1994/Dental procedure/Acupuncture/surgery/Endoscopic procedure	Fujian(2 months)/ Guangdong (23 years)	Husband(-) Son(-)
T14	26	Female	Guangdong	Multiple sexual partners /Dental procedure/Ear piercing/Shared toothbrush	Fujian/Guangdong	-
T15	47	Female	Hunan	-	-	-
T16	37	Female	Fujian	Ear piercing in 1997 in Fujian/Dental procedure/Surgery	Fujian	-
T17	21	Male	Guangdong	-	Guangdong	Elder brother(-)
T18	42	Male	Guangdong	-	-	-

Table S4. Summary of studies reporting HTLV seroprevalence among blood donors in China, including the present study.

No.	First author	Journal	Year	PMID / DOI	Region	Study period	No. screened	No. of screening-reactive	Screening-reactive rate (per 100,000)	No. of confirmed cases	Confirmed prevalence (per 100,000)	Counting unit
1	Present study	—	—	—	Shenzhen	2013-2022	512,200	203	39.63	18	3.51	Donors
2	Lin et al. [28]	Infect Dis Now	2024	PMID 38316362	Hangzhou, Ningbo, and Huzhou	2021.4 – 2022.3	2,760	1	36.23	—	—	Samples
3	Ji et al. [21]	Front Microbiol	2023	PMID 37920260	28 non-endemic provinces nationwide and Shenzhen	2019-2021	4,451,883	1276	28.66	50	1.12	Samples
4	Lee et al. [29]	Transfusion Medicine	2023	PMID 37209036	Taiwan, China	2009–2018	17,977,429	3707	20.62	739	4.11	Samples
5	Liao et al. [23]	PLOS Neglected Tropical Diseases	2021	PMID 33539355	21 cities in Guangdong	2016.3 –2018	3,262,271	1122	34.39	59	1.81	Donors

—	—	—	—	—	Guangzhou	2016.3–2018	1,014,424	—	—	11	1.08	Donors
—	—	—	—	—	Foshan	2016.3–2018	326,122	—	—	6	1.84	Donors
—	—	—	—	—	Dongguan	2016.3–2018	170,672	—	—	3	1.76	Donors
—	—	—	—	—	Huizhou	2016.3–2018	191,181	—	—	5	2.62	Donors
—	—	—	—	—	Zhuhai	2016.3–2018	59,378	—	—	1	1.68	Donors
—	—	—	—	—	Shenzhen	2016.3–2018	139,147	—	—	4	2.87	Donors
—	—	—	—	—	Jiangmen	2016.3–2018	118,185	—	—	2	1.69	Donors
—	—	—	—	—	Zhongshan	2016.3–2018	137,048	—	—	1	0.73	Donors
—	—	—	—	—	Zhaoqing	2016.3–2018	110,982	—	—	0	0	Donors
—	—	—	—	—	Chaozhou	2016.3–2018	24,645	—	—	0	0	Donors
—	—	—	—	—	Shantou	2016.3–2018	85,557	—	—	3	3.51	Donors
—	—	—	—	—	Shanwei	2016.3–2018	43,027	—	—	6	13.94	Donors
—	—	—	—	—	Jieyang	2016.3–2018	90,650	—	—	4	4.41	Donors

—	—	—	—	—	Yunfu	2016.3–2018	70,134	—	—	1	1.43	Donors
—	—	—	—	—	Yangjiang	2016.3–2018	64,965	—	—	0	0	Donors
—	—	—	—	—	Maoming	2016.3–2018	147,859	—	—	2	1.35	Donors
—	—	—	—	—	Zhanjiang	2016.3–2018	150,629	—	—	8	5.31	Donors
—	—	—	—	—	Shaoguan	2016.3–2018	154,946	—	—	1	0.65	Donors
—	—	—	—	—	Heyuan	2016.3–2018	42,440	—	—	0	0	Donors
—	—	—	—	—	Meizhou	2016.3–2018	94,374	—	—	1	1.06	Donors
—	—	—	—	—	Qingyuan	2016.3–2018	25,906	—	—	0	0	Donors
6	Chang et al. [19]	Retrovirology	2021	PMID 33413457	155 blood establishments in 30 provinces nationwide	2016–2018	8,411,469	4279	50.87	435	5.17	Donors
—	—	—	—	—	Fujian	2016–2018	681,564	939	137.77	247	36.24	Donors
—	—	—	—	—	Hunan	2016–2018	164,090	99	60.33	8	4.88	Donors

—	—	—	—	—	Zhejiang	2016–2018	1,384,135	460	33.23	56	4.05	Donors
—	—	—	—	—	Beijing	2016–2018	124,326	23	18.50	4	3.22	Donors
—	—	—	—	—	Jiangxi	2016–2018	240,310	107	44.53	7	2.91	Donors
—	—	—	—	—	Guangxi	2016–2018	171,951	112	65.13	5	2.91	Donors
—	—	—	—	—	Guangdong	2016–2018	2,755,108	1322	47.98	79	2.87	Donors
—	—	—	—	—	Sichuan	2016–2018	116,388	83	71.31	3	2.58	Donors
—	—	—	—	—	Xinjiang	2016–2018	38,965	35	89.82	1	2.57	Donors
—	—	—	—	—	Jilin	2016–2018	182,328	72	39.49	4	2.19	Donors
—	—	—	—	—	Heilongjiang	2016–2018	182,739	35	19.15	4	2.19	Donors
—	—	—	—	—	Hebei	2016–2018	202,170	140	69.25	4	1.98	Donors
—	—	—	—	—	Gansu	2016–2018	56,325	16	28.41	1	1.78	Donors
—	—	—	—	—	Jiangsu	2016–2018	294,997	63	21.36	4	1.36	Donors
—	—	—	—	—	Shaanxi	2016–2018	82,711	27	32.64	1	1.21	Donors

—	—	—	—	—	Guizhou	2016–2018	413,072	204	49.39	4	0.97	Donors
—	—	—	—	—	Yunnan	2016–2018	128,279	100	77.96	1	0.78	Donors
—	—	—	—	—	Henan	2016–2018	267,149	111	41.55	2	0.75	Donors
—	—	—	—	—	Anhui	2016–2018	142,604	68	47.68	0	0.00	Donors
—	—	—	—	—	Hainan	2016–2018	35,564	17	47.80	0	0.00	Donors
—	—	—	—	—	Hubei	2016–2018	138,704	38	27.40	0	0.00	Donors
—	—	—	—	—	Liaoning	2016–2018	98,876	26	26.30	0	0.00	Donors
—	—	—	—	—	Inner Mongolia	2016–2018	21,062	20	94.96	0	0.00	Donors
—	—	—	—	—	Ningxia	2016–2018	30,490	8	26.24	0	0.00	Donors
—	—	—	—	—	Shandong	2016–2018	251,019	94	37.45	0	0.00	Donors
—	—	—	—	—	Shanxi	2016–2018	55,651	24	43.13	0	0.00	Donors
—	—	—	—	—	Shanghai	2016–2018	69,479	11	15.83	0	0.00	Donors
—	—	—	—	—	Tianjin	2016–2018	51,394	19	36.97	0	0.00	Donors

—	—	—	—	—	Chongqing	2016–2018	30,019	6	19.99	0	0.00	Donors
7	Zhao et al. [22]	BMC Infect Dis	2020	PMID 32762656	Hebei	2018.11–2019.3	16,144	4	24.78	0	0.00	Donors
—	—	—	—	—	Changsha	2018.11–2019.3	15,164	3	19.78	0	0.00	Donors
—	—	—	—	—	Shenzhen	2018.11–2019.3	28,621	26	90.84	1	3.49	Donors
8	Chen et al. [26]	Expert Rev Hematol	2019	PMID 31220418	Nationwide (18 provinces and 4 municipalities)	1998–2017	1,420,079	—	—	—	16.90	Samples
9	Li et al. [25]	Transfusion	2019	PMID 30985961	20 blood centers nationwide	2016–2017	875,453	401	45.80	22	2.51	Samples
—	—	—	—	—	Xiamen Blood Center	2016–2017	108,176	—	—	12	11.09	Samples
—	—	—	—	—	Shenzhen Blood Center	2016–2017	208,647	—	—	6	2.88	Samples
—	—	—	—	—	Beijing Blood Center	2016–2017	63,385	—	—	2	3.16	Samples
—	—	—	—	—	Shenyang Blood Center	2016–2017	44,692	—	—	0	0.00	Samples

—	—	—	—	—	Dalian Blood Center	2016–2017	40,000	—	—	0	0.00	Samples
—	—	—	—	—	Changsha Blood Center	2016–2017	16,767	—	—	1	5.96	Samples
—	—	—	—	—	Nanjing Blood Center	2016–2017	101,923	—	—	1	0.98	Samples
—	—	—	—	—	Xuzhou Blood Center	2016–2017	50,289	—	—	0	0.00	Samples
—	—	—	—	—	Ma'anshan Blood Center	2016–2017	20,015	—	—	0	0.00	Samples
—	—	—	—	—	Bengbu Blood Center	2016–2017	20,021	—	—	0	0.00	Samples
—	—	—	—	—	Hefei Blood Center	2016–2017	20,540	—	—	0	0.00	Samples
—	—	—	—	—	Anqing Blood Center	2016–2017	20,322	—	—	0	0.00	Samples
—	—	—	—	—	Wuhu Blood Center	2016–2017	19,942	—	—	0	0.00	Samples
—	—	—	—	—	Suzhou Blood Center	2016–2017	51,216	—	—	0	0.00	Samples
—	—	—	—	—	Changzhi Blood Center	2016–2017	27,538	—	—	0	0.00	Samples
—	—	—	—	—	Haikou Blood Center	2016–2017	27,988	—	—	0	0.00	Samples
—	—	—	—	—	Nanchong Blood Center	2016–2017	4,560	—	—	0	0.00	Samples

—	—	—	—	—	Deyang Blood Center	2016–2017	3,000	—	—	0	0.00	Samples
—	—	—	—	—	Urumqi Blood Center	2016–2017	17,129	—	—	0	0.00	Samples
—	—	—	—	—	Yili Blood Center	2016–2017	9,303	—	—	0	0.00	Samples
10	Xie et al. [27]	PLoS Negl Trop Dis	2015	PMID 25830656	Xiamen	2004–2013	253,855	388	152.84	43	16.90	Samples
11	Wang et al. [24]	J Med Virol	2005	PMID 15977241	13 provinces nationwide	—	145,293	30	20.65	19	13.08	Samples
—	—	—	—	—	Fujian	—	34,800	26	74.71	0	0.00	Samples
—	—	—	—	—	Hainan	—	12,084	3	24.83	0	0.00	Samples
—	—	—	—	—	Tianjin	—	4,635	1	21.57	0	0.00	Samples
—	—	—	—	—	Jilin	—	8,900	0	0.00	0	0.00	Samples
—	—	—	—	—	Liaoning	—	18,300	0	0.00	0	0.00	Samples
—	—	—	—	—	Henan	—	14,266	0	0.00	0	0.00	Samples
—	—	—	—	—	Shandong	—	13,769	0	0.00	0	0.00	Samples
—	—	—	—	—	Jiangsu	—	9,500	0	0.00	0	0.00	Samples
—	—	—	—	—	Zhejiang	—	4,846	0	0.00	0	0.00	Samples
—	—	—	—	—	Hubei	—	8,800	0	0.00	0	0.00	Samples
—	—	—	—	—	Jiangxi	—	8,500	0	0.00	0	0.00	Samples
—	—	—	—	—	Xinjiang	—	1,820	0	0.00	0	0.00	Samples
—	—	—	—	—	Guangdong	—	5,073	0	0.00	0	0.00	Samples

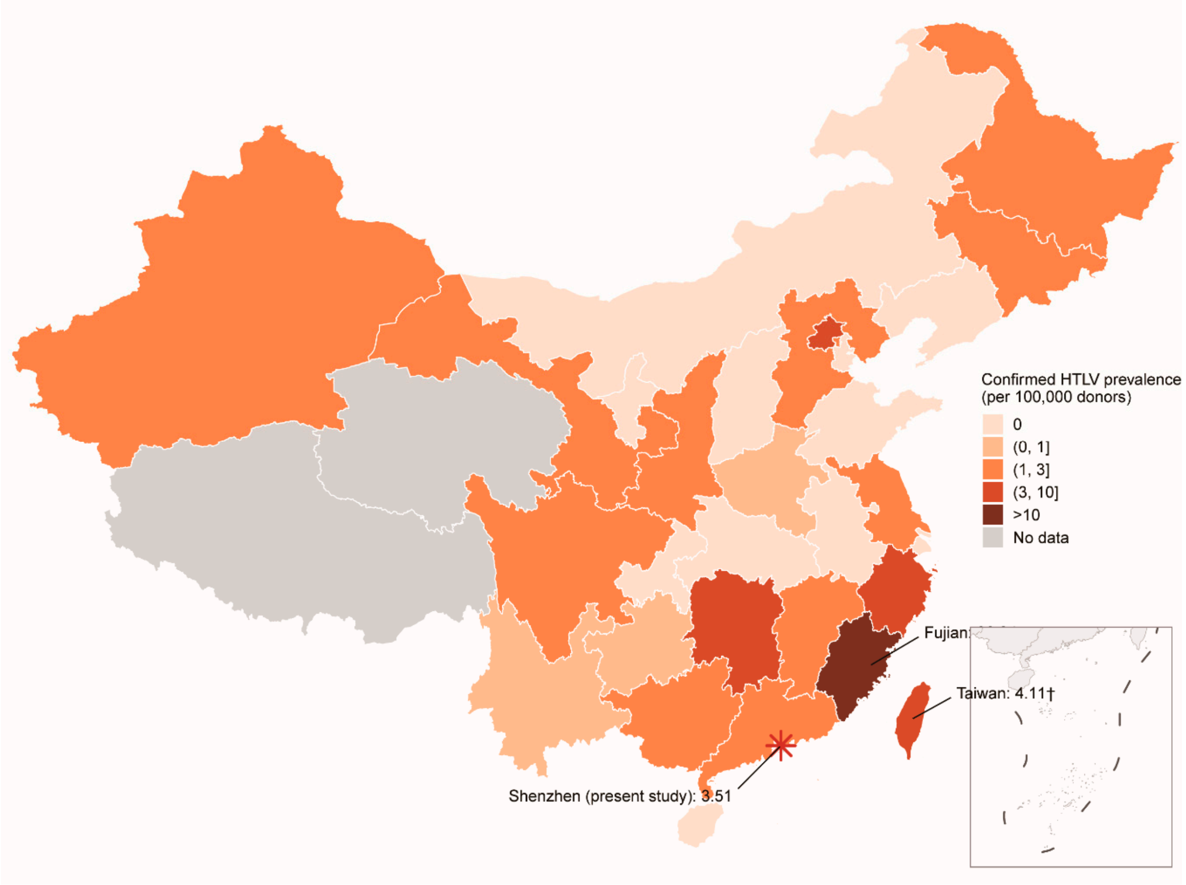

Figure S1. Geographical distribution of confirmed HTLV seroprevalence among blood donors in China. Province-level confirmed prevalences (per 100,000 donors, color scale) were derived from the nationwide survey by Chang et al. [19]. The estimate for Taiwan (4.11 per 100,000) was derived from Lee et al. [29]; † this estimate was calculated per donation rather than per donor (17,977,429 donations, 2008–2019). The red star marks Shenzhen (3.51 per 100,000 donors, present study). Values across studies are not directly comparable because of differences in screening and confirmatory algorithms.
